# The Incidence and Risk Factors for Adverse Drug Reactions Related to Tanreqing Injection: A Large Population-Based Study in China

**DOI:** 10.3389/fphar.2019.01523

**Published:** 2020-01-09

**Authors:** Xiao-Xiao Li, Lin Zhuo, Yan Zhang, Yi-Heng Yang, Hong Zhang, Si-Yan Zhan, Suo-Di Zhai

**Affiliations:** ^1^ Department of Pharmacy, Peking University Third Hospital, Beijing, China; ^2^ Department of Pharmacy Administration and Clinical Pharmacy, School of Pharmaceutical Sciences, Peking University, Beijing, China; ^3^ Research Center of Clinical Epidemiology, Peking University Third Hospital, Beijing, China; ^4^ Department of Epidemiology and Biostatistics, School of Public Health, Peking University, Beijing, China; ^5^ Technology and Development Center for TCM of China, State Administration of Traditional Chinese Medicine of the People’s Republic of China, Beijing, China

**Keywords:** drug safety, adverse drug reactions, pharmacovigilance, anaphylaxis, traditional Chinese medicine, off-label

## Abstract

**Background:** Tanreqing injection (TRQ) is a traditional Chinese medicine commonly used in China to treat pulmonary diseases presenting as phlegm-heat syndrome. Robust data on the safety of TRQ from real-world observational cohorts are currently lacking.

**Objective:** To evaluate as the incidence, type, and predictors of adverse events (AEs) and adverse drug reactions (ADRs) of TRQ in clinical practice in China.

**Methods:** We conducted a population-based cohort, multicenter study to evaluate the incidence, manifestation, outcomes, and risk factors of AEs and ADRs following TRQ use in China. Between April 2014 and May 2015 a total of 30,322 consecutive inpatients/emergency attendance patients from 90 hospitals across China administrated TRQ were followed-up for 7 days. Odds ratios (ORs) with 95% confidence intervals (CIs) were estimated using logistic regression to identify predictors of ADRs.

**Results:** The incidence of AEs and ADRs was 1.4 and 0.3%, respectively. Skin and subcutaneous tissue disorders were the most common ADRs. All ADRs were mild or moderate in severity, except for one serious case of anaphylactic reaction. The majority of ADRs (72.8%) occurred in the first 2 h after TRQ administration. Two-thirds of patients (66.1%) in the study were prescribed TRQ off-label, including infants aged ≤24 months. A history of food allergy (OR 4.50, 95% CI: 1.35–15.00), drug allergy (OR 2.77, 95% CI: 1.56–4.94), and fast infusion speed (off-label use) (OR 2.10, 95% CI: 1.27–3.50) were associated with an increased risk of ADRs.

**Conclusion:** TRQ is well tolerated in the general population, yet off-label use is prevalent. Efforts are required to educate prescribers to adhere to the drug label in order to minimize potential patient harm.

## Introduction

Tanreqing injection (TRQ) is a traditional Chinese medicine (TCM) consisting of five water-soluble herbals extracts: Radix *Scutellariae* [*Scutellaria baicalensis* Georgi (radix), 30%], bear bile powder (*Fel Ursi Selenarctos thibetanus* G. Cuvier *Ursus arctos* L., 9%), goral horn (*Naemorhedus goral* Hardwicke, 7%), *Flos Lonicerae* [*Lonicera japonica* Thunb (flos), 30%], and Fructus *Forsythiae* [*Forsythia suspense* (Thunb.) Vahl. (fructus), 24%], with baicalin, chlorogenic acid, ursodeoxycholic acid, and chenodeoxycholic acid as the major bioactive constituents (CFDA, 2012; Zhang et al., 2016; Li et al., 2019). Owing to its antibacterial, antiviral, and anti-inflammatory effects, it is widely used in China to treat diseases presenting as “phlegm-heart syndrome” (Li et al., 2010; Yang et al., 2018), including acute upper respiratory infections (Wang et al., 2016), pneumonia (Wang et al., 2011; Xiong et al., 2018), and acute exacerbation of chronic obstructive pulmonary disease (COPD) (Zhong et al., 2010). It was approved for use by the Chinese Food and drug Administration (CFDA) in 2003. Between 2009 and 2011, the National Centre of Adverse Drug Reaction (ADR) Monitoring, affiliated with the CFDA, received 4,899 ADR following use of TRQ, including 172 serious adverse events (SAEs) most of which were anaphylaxis. Forty five percent of SAEs occurred in children under 5 years of age or in adults aged over 60 years (the National Centre of ADR Monitoring, unpublished).

Plant based extracts and proteins from animal sources have been considered to be major allergens in TCM injections, and in 2010 an allergen-warning was added to the product label along with wording that the product is contraindicated in infants aged less than 24 months and pregnant women. Other contraindications stated on the label include hepatic, renal, or heart failure. In response to concerns that the multi-ingredient composition and low-quality standards in production may be factors related to TRQ ADRs (Ji et al., 2009; Zhang et al., 2014), in December 2011 the CFDA introduced a standardized process on active ingredient preparation to control the quality of TRQ production (Li et al., 2014; Yang, B. et al., 2017; Yang, Y. et al., 2017; Li, W. L. et al., 2017). As part of the post-marketing surveillance of TRQ, there is a need to gather safety data from large population-based observational cohorts, yet currently only a few small single-centered studies on this topic have been conducted (Zhong et al, 2010; Xie et al, 2014; Wang et al, 2018). Therefore we carried out a population-based study to evaluate the incidence, manifestation, outcomes and risk factors of ADRs and adverse events (AEs) following TRQ in clinical practice across China.

## Materials and Methods

### Study Design

We carried out a cohort study with prospective and retrospective components (ClinicalTrials.gov, identifier NCT02094638; Chinacohort.bjmu.edu.cn, identifier CCC2018062701). The study was conducted at 90 hospitals (55 general hospitals, 12 district/country hospitals, 9 specialized hospitals including women’s and children’s hospitals, and 14 TCM hospitals) within 27 provincial level administrative regions across China (see **Supplementary Figure A** and **Tables A**, **B** for details). Participating centers were selected based on sales of TRQ, scientific research capacity, and willingness to take part. The study was supported by State Administration of TCM of the People’s Republic of China (SATCM, project number: 2013ZX04) and approved by Peking University Third Hospital Ethics Committee with waiver informed consent (reference number: IRB00006761-2014009).

### Study Population

A total of 30,322 consecutive patients, either inpatients or those attending a hospital emergency department, who were administered TRQ for the first time between January 2014 and May 2015 were identified. This included 2,743 patients who were newly prescribed TRQ from January to March of 2014 and who belonged to a retrospective cohort, and 27,579 patients identified between April and May 2015 that formed a prospective cohort. In view of the short half-live of TRQ (Liu et al., 2013; Li et al., 2018), every patient involved was followed up for 7 days after either TRQ was discontinued or the date of hospital discharged.

### Data Sources, Collection, and Quality Control

**Figure 1:**
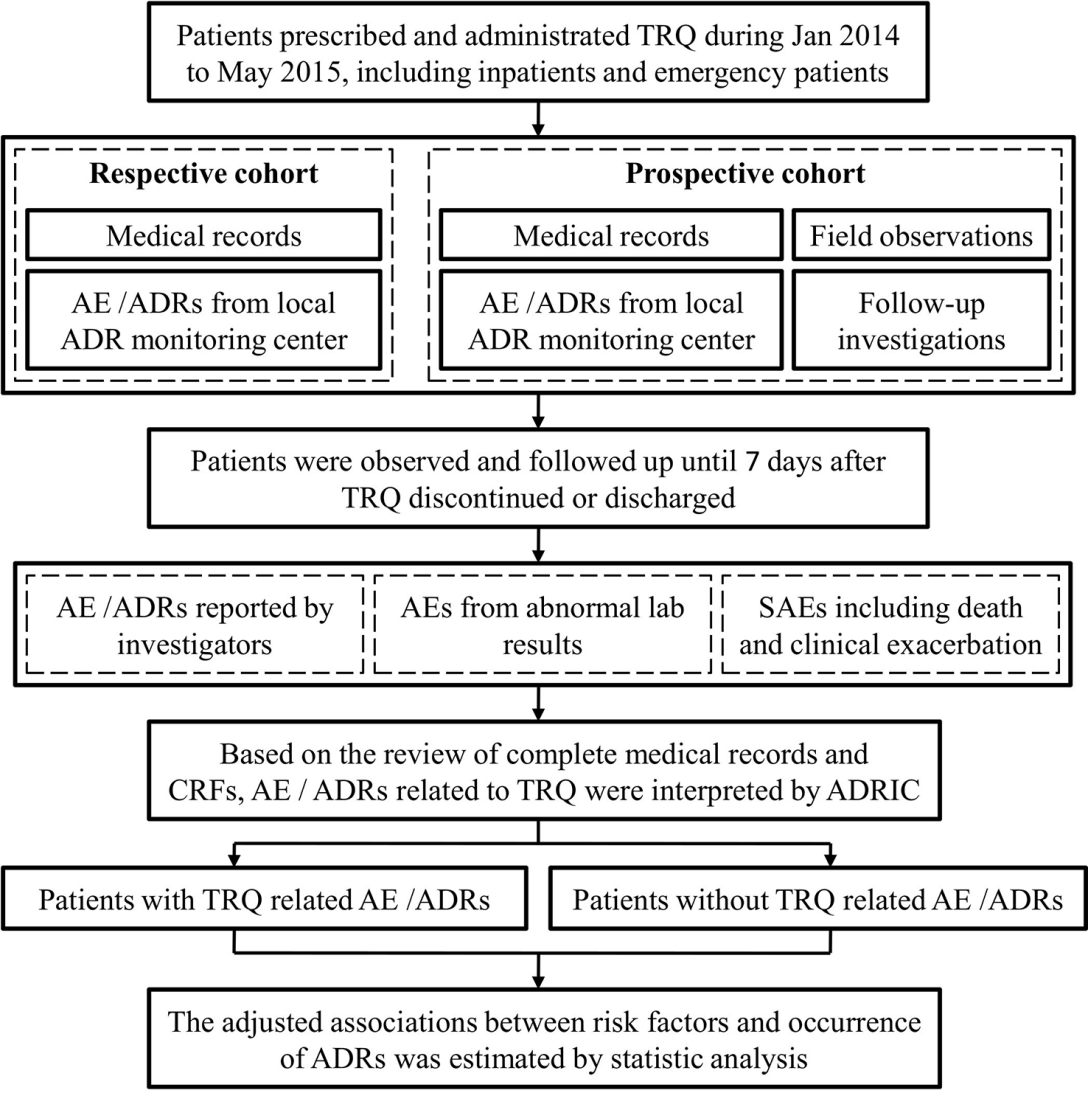
Flow chart for adverse event (AE)/adverse drug reactions (ADRs) identification. The figure depicts the flow of AE/ADR identification from data collected from field observations and follow-up investigations in respective and prospective cohort. TRQ, Tanreqing injection; SAEs, serious adverse events; ADRIC, ADR interpretation committee.

The methods of data collection are summarized in **Figure 1**. Two local investigators (predominantly pharmacists) in each study site were responsible for the data collection, which included a details on all prescriptions written for TRQ issued from the central hospital pharmacy (data collected daily), demographics, medical history (including history of allergy), diagnoses (including Chinese medical diagnoses), laboratory test results, medication use, details of TRQ administration, and AEs. Historical records, including medical records and reported ADRs were collected for patients in the retrospective and prospective cohort, while data from follow-up investigations were collected in the prospective cohort. Adverse events were also identified from the medical records of patients who died or who had disease exacerbation or abnormal laboratory test results.

Local investigators submitted data *via* a secure and password-protected web-based electronic data capture system (EDC, http://182.92.8.232/commedc/). International Classification of Diseases-10, Medical Dictionary for Regulatory Activities, and World Health Organization-Anatomical Therapeutic Chemical codes were used as the data dictionary in the EDC for standardizing entries relating to medical history and diagnoses, AEs, and medications. To minimize transcription errors, local hospital information system (if authorized) was linked to the electronic data by matching on a series of identifiers for downloading basic information in the case report form (CRF), including demographics, laboratory test results, and economic outcomes. Each patient was assigned a unique ID and the submitted data were checked three times—by the second local investigator, by a clinical research associate, and by a data administrator. Any CRF with errors was returned to the first investigator inputting the data who subsequently revised the entry. The department of technology and development center for TCM, SATCM served as the point of data collection, coordination center, and de-identification. Data analysis was carried out at the Department of Epidemiology and Biostatistics, School of Public Health, Peking University.

### Assessment of Adverse Events and Adverse Drug Reactions

A multidisciplinary ADR interpretation committee (ADRIC) assessed and interpreted all ADRs in accordance with the WHO Collaborating Centre for International Drug Monitoring (WHO-UMC) causality assessment system. The relationship between the reported ADRs and TRQ was categorized as certain, probable, possible, unlikely, conditional/unclassified, or unassessable/unclassifiable. Adverse events were graded as mild, moderate, and severe (Edwards and Aronson, 2000). A serious adverse event (SAE) was defined as any untoward medical occurrence that resulted in death, required hospital admission, prolonged an existing hospital stay, or was life threatening (Edwards and Aronson, 2000). A new ADR was described as either an unlabeled ADR or an ADR where severity is insufficiently described on the current drug label, as appropriate. Anaphylaxis was determined based on the criteria recommended by the National Institute of Allergy and Infectious Diseases, the Food Allergy and Anaphylaxis Network, and the European Academy of Allergy and Clinical Immunology (Muraro et al., 2014; Sampson et al., 2016). The incidence of TRQ-related ADRs was the primary outcome measure. The incidence of AEs, outcomes of risk factors for TRQ-related ADRs were secondary outcomes. Indications for TRQ use and off-label prescribing were also described.

### Statistics Analysis

PASS 11.0.7 was used to calculate the sample size. Based on an incidence of ADRs (0.14%) in the phase I to IV trials (Shanghai Kaibao Pharmaceutical Co., LTD, unpublished; Shen et al, 2007), and an expected 20% loss-to-follow-up, we estimated that 30,400 patients would be needed to observe an ADR incidence of less than 0.1% with a probability of 95%. Categorical variables were described using frequency counts and percentages, and continuous variables were described using medians with interquartile range (IQR). Multivariable non-conditional logistic regression model was then used to estimate the association between potential risk factors and occurrence of ADRs related to TRQ by calculating odds ratios (OR) with 95% confidence intervals (CIs). All *p* values were two-sided. SAS version 9.2 was used for all statistical analyses.

## Results

### Baseline Characteristics

Of the 30,322 consecutive patients administrated TRQ between January 2014 and May 2015, 22,062 (72.8%) were from general hospitals and 3,475 (11.5%) were from TCM hospitals. Baseline characteristics of the study population are shown in **Table 1** and **Supplementary Figure B**. There were 11,621 (38.33%) females and the median age was 57 years (IQR 26, 72); 41% of patients were aged at least 60 years. A total of 2,326 (7.7%) patients had a history of food or drug allergy or other allergic disease. The median length of hospital stay was 11 days. TRQ-related costs accounted for 5.1% of the total medical costs with most covered by health insurance.

**Table 1:** Baseline characteristics of the study population. Data are n (%) unless otherwise stated.

Patient characteristic	Patients, N = 30,322
**Demographics and lifestyle factors**	
Median age (IQR), years	57 (26, 72)
<18	6,500 (21.4)
≥60	13,061 (41.1)
Female	11,621 (38.3)
Han ethnicity	29,589 (97.6)
***History of allergy*** ^**a**^	2,326 (7.7)
Food allergy	174 (0.6)
Drug allergy	1,969 (6.5)
Allergic disease	292 (1.0)
***Lifestyle***	
Current smoking	7,447 (24.6)
Current alcohol drinker	4,898 (16.2)
**Hospitalization factors**	
Median length of stay (IQR), days	11 (8, 18)
Death in hospital	239 (0.8)
***Hospital department ^c^***	
Pneumology	7,461 (24.6)
Pediatrics	4,794 (15.8)
Infectious disease	1,613 (5.3)
General surgery	1,420 (4.7)
Emergency	963 (3.2)
***TRQ related diagnosis (ICD-10 codes)*^b,c^**	
Pneumonia (J18)	9,760 (32.2)
Acute upper respiratory infections (J00-J06)	4,937 (16.3)
Chronic obstructive pulmonary disease (J44.9)	2,609 (8.6)
Acute bronchitis (J20)	2,500 (8.2)
Encounter for prophylactic surgery (Z40) ^e^	1,851 (6.1)
Chronic bronchitis (J42)	1,244 (4.1)
*Enteroviral* vesicular stomatitis with exanthema (B08.4)	899 (3.0)
***Combined diseases (ICD–10 codes)*^b,d^**	
Hypertensive diseases (I10–I16)	5,308 (17.5)
Atherosclerotic heart disease of native coronary artery (I25.1)	2,278 (7.5)
Type 2 diabetes mellitus (E11)	2,169 (7.2)
Cerebrovascular diseases (I60-I69)	1,442 (4.8)
Symptoms and signs involving the digestive system and abdomen (R10–R19)	1,307 (4.3)
***Characteristic of TRQ administration*^f^**	
Median dosage (IQR), ml/d	20 (20, 30)
Median duration of therapy (IQR), days	6 (4, 9)
Median preparation concentration of TRQ (IQR), %	8 (8, 12)
Median duration from preparation to the administration of TRQ (IQR), minutes	30 (15, 30)
Median (Q1, Q3) infusion rate (IQR), drops/min ^g^	40 (35, 50)

a A patient could have ≥2 different allergy history.

b Only variables accounting for ≥2.9% patients are shown.

c A patient could have ≥2 different TRQ-related diagnoses.

d A patient could have ≥2 different types of combined disease.

e Z40 was applicable to describe infection prevention for surgery.

f The labeled recommended intravenous dosage of TRQ for adults is 20 ml for each dose once a day into 250~500 ml of 5% GS or 0.9% NS and doses up to a maximum of 40 ml for severe cases. The labeled recommendation for pediatric patients is 0.3~0.5 ml/kg up to a maximum of 20 ml per dose once a day through intravenous infusion.

g Median (IQR) infusion rate (ml/h) was 120 (105, 150) using needles of 0.55*19 mm or 0.7*24 mm. ICD, International Classification of Diseases; IQR, interquartile range; TRQ, Tanreqing injection.

Overall, TRQ was administered for 32,883 different indications (a patient could have more than one different indication recorded). Infections and respiratory diseases were the most common accounting for 72.4% of indications, with acute upper respiratory tract pneumonia, COPD, bronchitis (acute or chronic), and hand, foot, and mouth disease being the most frequent indications. Most prescriptions for TRQ were made in accordance with the product label, including dosage (maximal dose being 20 ml for children, and 40 ml for adults), preparation concentration (≤10%), diluents (5% glucose or 0.9% normal saline), intravenous infusion mode of administration, and infusion rate (30–60 drops/min). However, a total of 20,058 (66.1%) patients were administered TRQ as off-label use (see **Supplementary Table C**). Of these, 1,851 (6.1%) patients were prescribed TRQ for pre-surgical infection prophylaxis, and 1,165 patients (3.8%) were aged ≤24 months, i.e., contraindicated. There were 459 patients (1.5%) with other contraindications as stated on the product label: hepatic failure (n = 138, 0.5%), renal failure (n = 292, 1.0%), both hepatic and renal failure (n = 17, 0.06%), and severe pulmonary heart disease with heart failure (n = 12, 0.04%). Also, 11,807 (38.9%) patients were administered TRQ with a preparation concentration of >10%, 2,058 (6.8%) patients were prescribed diluents beyond 5% glucose or 0.9% normal saline, and six patients (0.02%) were administered TRQ by aerosol inhalation. There were 30,055 (71.9%) patients who received TRQ concomitantly with other treatments such as systemic antibiotics, blood substitutes and perfusion solutions, and cough and cold preparations (see **Supplementary Table D**). A total of 173 (0.6%) patients were administered TRQ with other drugs within a same bottle/bag, which is another prohibition of use on the product label. In nine (0.03%) of these patients, the chemical reaction from co-administration was visually evident, showing up as cloudy white precipitate when TRQ was combined with doxofylline injection (three patients), levofloxacin lactate injection (three patients), moxifloxacin hydrochloride injection (two patients), or ornidazole and sodium chloride injection (one patient).

### Incidence and Casualty Assessment of Adverse Events and Adverse Drug Reactions

A total of 434/30,322 patients (1.4%) experienced an AE, of whom 263 experienced a SAE. As shown in **Table 2**, according to the WHO-UMC causality criteria, 81 patients (0.3%) were classified to have a TRQ-related ADR (one was deemed as certain, 35 probable and 45 possible). The most common TRQ-related ADRs were rash (40.7%), chest discomfort (7.4%), and nausea (6.2%). Only one patient experienced a serious TRQ-ADR—a 65 years old female who presented with anaphylactic reaction with the involvement of laryngeal edema 13 min after the administration of TRQ alone, and who recovered following treatment with promethazine and dexamethasone. All other TRQ-related ADRs were either mild (n = 59, 72.8%) or moderate (n = 21 patients, 25.9%). Twelve new symptoms (“new ADRs”) occurred among 22 patients, with chest discomfort accounting for just over a quarter (26.1%). There was little evidence of a difference in the incidence of ADRs between the patients in the prospective cohort (n = 76, 0.3%) and those in the retrospective cohort (n = 5, 0.2%, *p* = 0.37) or between patients in western medicine hospitals (n = 76, 0.3%) and those in TCM hospitals (n = 5, 0.1%, *p* = 0.14).

**Table 2:** Casualty assessment of adverse events. Data are n (%).

Casualty assessment^a^	AEs N = 434	
	All AEs	SAEs
Certain	1 (0.003)	0
Probable	35 (0.1)	1 (0.003)
Possible	45 (0.1)	0
Unlikely	329 (1.1)	240 (0.8)
Conditional	9 (0.03)	9 (0.03)
Unassessable	15 (0.05)	13 (0.04)
Total	434 (1.4)	263 (0.9)

a The estimation of relationship likelihood between AEs and TRQ were categorized based on WHO-UMC causality assessment system.

AE, adverse event; SAE, serious adverse event; TRQ, Tanreqing injection; WHO-UMC, World Health Organization-Uppsala Monitoring Centre.

### Characteristics of Adverse Drug Reactions

The frequency of TRQ-related ADRs according to the time of onset is shown in **Table 3**. The time interval between TRQ administration and ADR onset ranged between 5 min and 10 days. Among patients experiencing a TRQ-related ADR, the majority (n = 59, 72.8%) occurred within the first 2 h of drug administration. All patients who experienced an ADR saw their condition either improved (n = 45, 55.56%) or completely resolved (n = 36, 44.44%) after taking management measures (n = 79, 97.5%) or within time (n = 2, 2.5%). Management measures included TRQ withdrawal (n = 39, 48.1%), reintroducing of essential medicines (n = 22, 27.2%), both of these (n = 17, 21.0%), reduction in drip speed (n = 1, 1.2%). Recovery time varied from less than an hour (n = 27, 33.3%) to more than 24 h (n = 34, 42.0%).

**Table 3:** Frequency of Tanreqing injection-related adverse drug reactions according to the time of onset. Data are n (‰).

**ADRs**	**The time interval between TRQ administration and the onset of ADRs (hours)**				**Total patients (***n*** = 81)**
	≤0.5 **(n = 25)**	**0.5**–**2 (n = 34)**	**2**–**24 (n = 16)**	>**24 (** ***n*** **= 6)**	
***Cardiac disorders***						
Palpitations	2 (0.07)	0	2 (0.07)	0	4 (0.1)
***Eye disorders***						
Periorbital edema^b^	0	1 (0.03)	0	0	1 (0.03)
***Gastrointestinal disorders***						
Abdominal distension	0	1 (0.03)	0	0	1 (0.03)
Diarrhea	0	0	2 (0.07)	0	2 (0.07)
Nausea	4 (0.01)	1 (0.03)	0	0	5 (0.2)
Vomiting	0	2 (0.07)	0	0	2 (0.07)
***General disorders and administration site conditions***						
Asthenia^b^	0	1 (0.03)	0	0	1 (0.03)
Chest discomfort^b^	3 (0.10)	2(0.07)	1 (0.03)	0	6 (0.2)
Chills	3 (0.10)	0	1 (0.03)	0	4 (0.1)
Edema^b^	0	0	1 (0.03)	0	1 (0.03)
Hyperpyrexia^b^	1(0.03)	1(0.03)	0	0	2 (0.07)
Infusion site discomfort	2 (0.07)	0	0	0	2 (0.07)
Pyrexia	1 (0.03)	2 (0.07)	0	0	3 (0.1)
***Infections and infestations***						
Laryngitis^b^	0	0	0	1 (0.03)	1 (0.03)
***Investigations***						
Blood pressure decrease ^b^	1 (0.03)	0	0	0	1 (0.03)
Hepatic enzyme increased^b^	0	0	1 (0.03)	1 (0.03)	2 (0.07)
***Nervous system disorders***						
Dizziness	1 (0.03)	1 (0.03)	0	0	2 (0.07)
***Renal and urinary disorders***						
Hematuria ^b^	0	0	2 (0.07)	0	2 (0.07)
***Respiratory, thoracic, and mediastinal disorders***						
Cough^b^	1 (0.03)	0	0	0	1 (0.03)
Dysphonia^b^	0	1 (0.03)	0	0	1 (0.03)
Dyspnoea^b^	2 (0.07)	2 (0.07)	0	0	4 (0.1)
Laryngeal edema ^c^	1 (0.03)	0	0	0	1 (0.03)
Tachypnoea	0	1 (0.03)	0	0	1 (0.03)
***Skin and subcutaneous tissue disorders***						
Pruritus	0	1 (0.03)	0	1 (0.03)	3 (0.1)
Rash	10 (0.3)	16 (0.5)	4 (0.1)	3 (0.10)	33 (1.1)
Urticaria	0	1 (0.03)	2 (0.07)	0	3 (0.1)
***Vascular disorders***						
Flush	2 (0.07)	4 (0.01)	1 (0.03)	0	7 (0.02)
Phlebitis	0	3 (0.1)	1 (0.03)	0	4 (0.01)
**Total**	34 (1.1)	42 (1.4)	18 (0.6)	6 (0.2)	100 (3.3)

a ADRs are presented as individual symptoms and system organ class, based on the MedDRA classification. Frequency was calculated as number/30,322*1,000‰.

b New ADRs.

c A 65 years old female who presented with anaphylactic reaction with the involvement of laryngeal edema 13 min after the administration of TRQ alone.

ADR, adverse drug reaction; TRQ, Tanreqing injection; MedDRA, Medical Dictionary for Regulatory Activities.

The characteristics of ADRs among patients in the retrospective cohort were similar to those in the prospective cohort. Five ADRs occurred in five patients in the retrospective cohort: skin and subcutaneous tissue disorders such as rash (n = 2, 0.07%), pruritus (n = 1, 0.04%), urticaria (n = 1, 0.04%), and palpitations (n = 1, 0.04%). There was one patient in the retrospective cohort whose ADR (rash) that occurred in the first 2 h after TRQ administration, while three cases occurred between 2 and 24 h after TRQ administration. Only one ADR (pruritus) occurred 10 days after TRQ administration. All five patients in the retrospective cohort who experienced an ADR improved after TRQ withdrawal.

### Risk Factors for Tanreqing Injection-Related Adverse Drug Reactions

**Figure 2:**
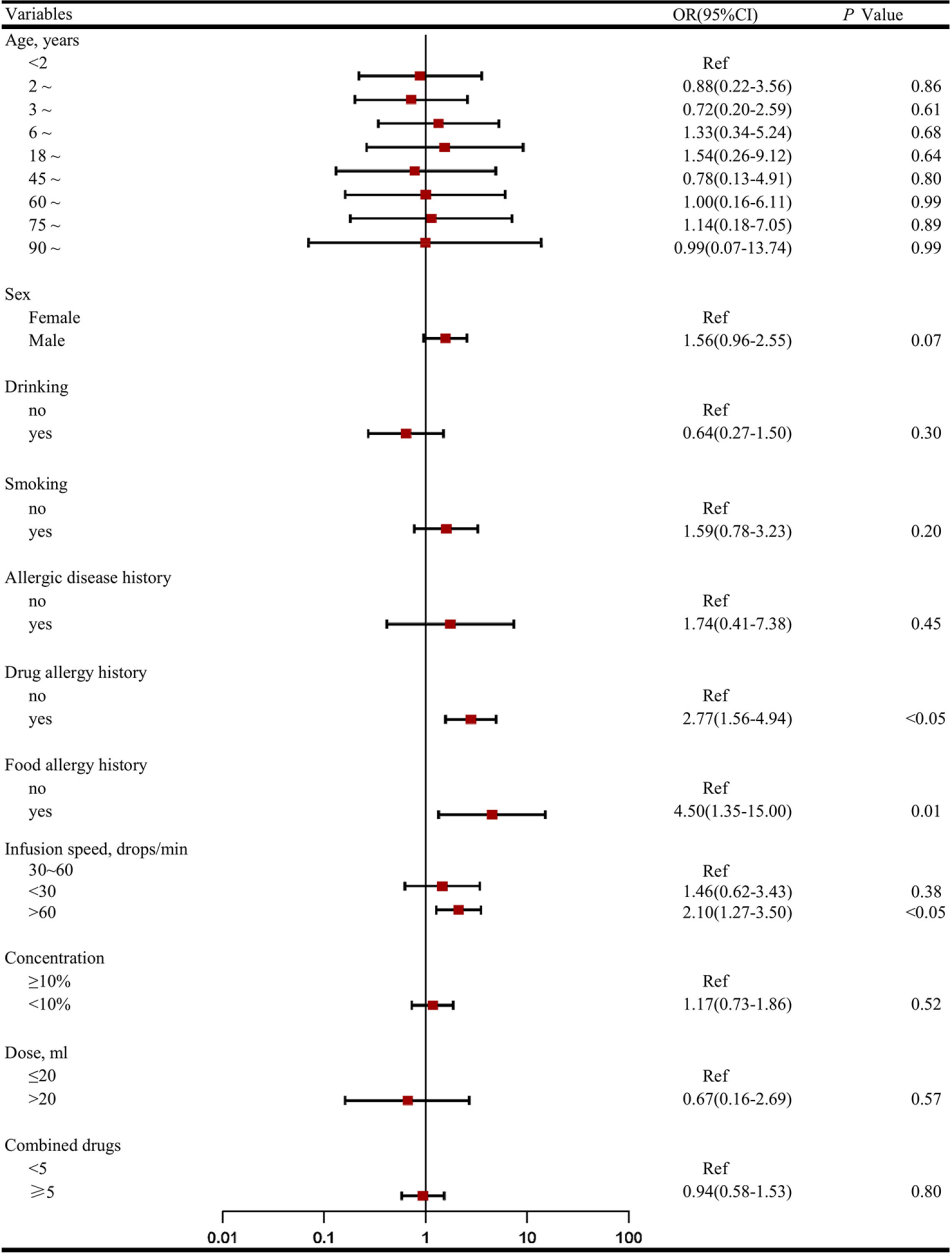
Factors associated with the incidence of adverse drug reactions (ADRs)^a^. **(A)** The odds ratio (OR) for demographic characters (age, sex, food/drug allergy, allergic disease history, habit of smoking/drinking) and clinical characteristics characters (infusion speed, concentration, dose, combined drugs) were compared with patients aged <2 years, female, no food/drug allergy history, no allergic disease history, no habit of smoking/drinking, standard treatment (infusion speed 30~60 drops/min, concentration < 10%, dose < 20 ml) and combined drugs < 5. **(B)** Considering most ADRs occurred in the first 2 h after administration and almost half of them withdrawal Tanreqing injection, we dropped therapy duration as risk factor in logistic regression model.

As shown in **Figure 2**, factors associated with an increased risk of TRQ related ADRs were having history of food allergy (OR 4.50, 95% CI: 1.35–15.00), drug allergy (OR 2.77, 95% CI: 1.56–4.94), and an infusion speed of >60 drops/min [*vs.* 30–60 drops/min; OR 2.10, 95% CI (1.27– 3.50)].

## Discussion

Our study is the first large-scale population-based study to evaluate the safety of TRQ among patients in real-world clinical practice in China, providing valuable information for patients, physicians, and drug safety regulators. We found that the incidence of AEs and ADRs following TRQ was low at 1.4 and 0.3%, respectively, with most non-serious in severity and with the majority occurring within a couple of hours of drug administration. This suggests that TRQ is well-tolerated. However, we also found that two-thirds of patients administered TRQ received it not according to label, with nearly 6% of off-label use being in infants aged less than 24 months (1,165 in 20,058 patients). Other off-label use related to non-approved indications, dosage, preparation concentration, diluents, mode of administration way, or infusion rate. Off-label use has been reported in a previous literature review of small studies of TRQ use (Wang et al, 2019), including to children people with a history of drug allergy, and with an infusion speed of >60 drops/min also reported. As suspected, we found that a history of allergy, be it food or drug allergy, was found to be the strongest predictor of TRQ-ADRs in line with the caution on the product label for use in these patients. Fast infusion speed of >60 drops/min was also identified as a predictor or TRQ-related ADR, and the incidence of TRQ-ADRs among children reported in our previous literature (Li, X. X. et al., 2017) was higher incidence than in the general population (0.4 *vs.* 0.3%).

In our study, the most common TRQ-related ADRs related to the skin and appendages, and the majority ADRs occurred within the first 2 h of TRQ administration, being similar to the results reported in previous studies (Shen et al., 2007; Li et al, 2010; Zhong et al., 2010; Xie et al., 2014; Wang et al., 2016; Wang et al, 2019). However, it is important to note that the incidences of TRQ-related AEs and ADRs seen in our study are higher than those seen in TRQ clinical trials (0.3% *vs.* 0.1~0.14%) (Shen et al., 2007; Li et al, 2010; Zhong et al., 2010; Xie et al., 2014; Wang et al., 2016), highlighting the importance of post-marketing surveillance. Our study included a broad range of patients, including those with a history of allergy, infants, and the elderly who were excluded from the TRQ clinical trials (Shanghai Kaibao Pharmaceutical Co., LTD, unpublished; Shen et al., 2007). Although only one patient in our study suffered anaphylaxis, this suggests that prescribers should be aware that this severe adverse reaction is possible, and a management practices should be in place in case of this outcome.

Our study has several strengths. In addition to the large sample size, the broad study population was drawn from a large number of hospitals, including those of different specialties and with a wide geographical spread across China. Our results will therefore be more generalizable to the individuals prescribed TRQ in the general population of China than those from small single-centered studies. Investigators used multiple data source/lines of investigation to identify AEs, which were reviewed and interpreted by the ADRIC based on patients’ complete patient medical records. Another strength of our study is the involvement of pharmacists as principle investigators at the study sites, which has been a feature of few other TCM post-marketing studies (Yan et al., 2017). Pharmacists are able to employ their expertise around prescribing issues and pharmacovigilance, and may have access to hospital pharmacy data unlike some other qualified health professionals.

Limitations of study should also be acknowledged. Firstly, our study population included patients in a retrospective cohort for whom some prospectively collected safety data, for example, patient self-reported AEs, could not be obtained. This may have lead to information bias and the possibility of underestimating AE incidence, although we found no evidence of a difference in the incidence of ADRs among patients in the retrospective and prospective cohorts. Secondly, our study included inpatient and emergency patients treated with TRQ and not individuals who were treated outside of this setting. A recent study reported that over 50% of outpatients were prescribed with an injection-administered medication, other than oral medications, in rural township health centers in China (Jiang et al., 2012). There may therefore have been some selection bias toward a study population slightly less healthy than the true population prescribed TRQ, and this might lead to an overestimation of the true incidence of TRQ-related ADRs. Conversely, and thirdly, follow-up in our study was limited to 7 days and therefore later-onset AEs/ADRs will not have been documented, leading to an underestimation of AE/ADR incidence. Fourthly, AE data from laboratory investigations in our study were based on those included in the TRQ phase III and IV clinical trials (*Shanghai Kaibao Pharmaceutical Co., LTD, unpublished*; Shen et al., 2007), but focused on those from routine blood, hepatic and renal function tests, hepatic and results from other clinical inspection were not investigated. This too may have led to an underestimation of AE/ADR incidence. Lastly, we were unable to report on the specific type of allergic AEs/ADRs TRQ based on the recorded data.

In conclusion, our results suggest that the incidence AEs and ADRs among patients treated with TRQ in clinical practice in China is higher than that seen previously in TRQ clinical trials and that off-label use is highly prevalent. Further data from other well-designed population-based studies in China are warranted to compare with findings from our study, and to help guide physicians and regulators as to whether a review of the current recommendations for TRQ is necessary. Efforts should be made to help TRQ prescribers adhere to the prescribing recommendations on the drug label in order to minimize avoidable patient harm.

## Author's Note

Findings from this study were presented in poster format at the 76^th^ International Pharmaceutical Federation’s World Congress of Pharmacy and Pharmaceutical Sciences, Buenos Aires, Argentina from August 28 -September 1, 2016, 32^nd^ International Conference on Pharmacoepidemiology & Therapeutic Risk Management, Dublin, Ireland, August 25–28, 2016.

## Data Availability Statement

All datasets generated for this study are included in the article/**Supplementary Material**.

## Ethics Statement

The studies involving human participants were reviewed and approved by Peking University Third Hospital Ethics Committee with a waiver for informed consent (reference number: IRB00006761- 2014009). Written informed consent from the participants’ legal guardian/next of kin was not required to participate in this study in accordance with the national legislation and the institutional requirements.

## Author Contributions

All authors took part in the final version for submission and accept overall accountability for the accuracy and integrity of the manuscript. S-DZ developed and conceptualized the topic. S-DZ, S-YZ, Y-HY, and HZ designed the methodology. X-XL, LZ, and YZ administrated the project, under supervision conducted by S-DZ and S-YZ. X-XL and YZ conducted the investigation, in cooperation with LZ, who carried out data curation and formal analysis. X-XL and LZ developed the visualization and writing of the original draft.

## Funding

This work was supported by the State Administration of Traditional Chinese Medicine of the People’s Republic of China (grant number: 2013ZX04).

## Conflict of Interest

X-XL is supported by National Key Clinical Specialty Discipline Construction Project 56495-04 and by Peking University Third Hospital, funded through State Administration of Traditional Chinese Medicine of the People’s Republic of China 2013ZX04. LZ is supported by School of Public Health, Peking University, funded through State Administration of Traditional Chinese Medicine of the People’s Republic of China 2013ZX04. YZ is supported by State Administration of Traditional Chinese Medicine of the People’s Republic of China 2013ZX03 and 2013ZX04. Y-HY is supported by National Key Clinical Specialty Discipline Construction Project 56495-04 and by Peking University Third Hospital, funded through State Administration of Traditional Chinese Medicine of the People’s Republic of China 2013ZX03 and 2013ZX04. HZ is supported by State Administration of Traditional Chinese Medicine of the People’s Republic of China 2013ZX03 and 2013ZX04. S-YZ is supported by School of Public Health, Peking University, funded through State Administration of Traditional Chinese Medicine of the People’s Republic of China 2013ZX03 and 2013ZX04. S-DZ is supported by National Key Clinical Specialty Discipline Construction Project 56495-04 and by Peking University Third Hospital, funded through SATCM 2013ZX03 and 2013ZX04.

## Abbreviations

ADRIC, ADR interpretation committee; ADRs, Adverse drug reactions; AEs, Adverse events; CRF, Case report forms; CFDA, China Food and Drug Administration; COPD, Chronic obstructive pulmonary disease; CRA, Clinical research associate; CIs, Confidence intervals; EDC, Electronic data capture; EVSE, *Enteroviral* vesicular stomatitis with exanthema; OR, Odds ratios; TReQS, Post-Marketing Surveillance of the Tanreqing Injection: a Real-World Study; SAEs, Serious adverse events; SATCM, State Administration of Traditional Chinese Medicine of the People’s Republic of China; TRQ, Tanreqing injection; TCM, Traditional Chinese medicine. IQR, interquartile range; WHO-UMC, World Health Organization - Uppsala Monitoring Centre.
