# Supplementary material for: The Incidence and Risk Factors for Adverse Drug Reactions Related to Tanreqing Injection: A Large Population-Based Study in China
Source: Front Pharmacol. 2020 Jan 9;10:1523. doi: 10.3389/fphar.2019.01523 (PMC6962140; doi:10.3389/fphar.2019.01523)

Supplementary Table A. Characteristics of surveillance sites.

Variables	Patients, N=30,322 n (%)	Study sites, N=90 n (%)
**Hospital type**		
General	22,062 (72.8)	55 (61.1)
Traditional Chinese medicine	3475 (11.5)	14 (15.6)
District or county	2437 (8.0)	12 (13.3)
Specialized	2348 (7.8)	9 (10.0)
**Hospital distribution**		
North China	8741(28.8)	26 (28.9)
West China	8684 (28.6)	21 (23.3)
Central China	5684 (18.7)	12 (13.3)
East China	4286 (14.1)	17 (18.9)
South China	1380 (4.6)	4 (4.4)
Northwest China	1099 (3.6)	7 (7.8)
Northeast China	448 (1.5)	3 (3.3)

Supplementary Table B. The distribution of surveillance sites and sample sizes.

Hospital	Area [a]	Provincial-level administrative region	City	types [b]	Numbers of patients enrolled in a retrospective cohort	Numbers of patients enrolled in a prospective cohort	AEs (R/P)[c]	ADRs (R/P)[c]
Beijing Luhe Hospital, Capital Medical University	NC	Beijing	Beijing	G	/	101	10	0
Peking University Third Hospital	NC	Beijing	Beijing	G	/	50	11	1
Beijing Geriatric Hospital	NC	Beijing	Beijing	G	/	66	0	0
The 263rd Hospital of Chinese People's Liberation Army	NC	Beijing	Beijing	D/C	/	304	9	1
Fengtai district Hospital of Chinese and Western medicine, Beijing	NC	Beijing	Beijing	T	/	99	3	0
The 464th Hospital of Chinese People's Liberation Army	NC	Tianjin	Tianjin	G	98	335	8/2	0
The 254th Hospital of Chinese People's Liberation Army	NC	Tianjin	Tianjin	G	86	64	0	0
First Hospital of Handan City	NC	Hebei	Handan	G	732	1792	55/33	5/2
Bethune International Peace Hospital of the Chinese PLA	NC	Hebei	Shijiazhuang	G	/	513	0	0
People's Hospital of Cangzhou	NC	Hebei	Cangzhou	G	440	553	3/1	0
The First Hospital of Qinhuangdao	NC	Hebei	Qinhuangdao	G	333	1178	59/9	0/2
The Second Hospital of Hebei Medical University	NC	Hebei	Shijiazhuang	G	/	306	15	3
People's Hospital of Rongcheng	NC	Hebei	Baoding	D/C	/	131	1	0
Hebei Zhaoxian People's Hospital	NC	Hebei	Shijiazhuang	D/C	/	102	0	0
Hejian Municipal People's Hospital	NC	Hebei	Hejian	D/C	/	229	0	0
Xinle Municipal Hospital of Hebei	NC	Hebei	Xinle	D/C	/	271	0	0
The Second Hospital of Wulanchabu City	NC	Neimenggu	Wulanchabu	D/C	/	153	2	0
The Third Affiliated Hospital of Inner Mongolia Medical College	NC	Neimenggu	Baotou	G	/	142	0	0
The fourth Hospital of Inner Mongolia	NC	Neimenggu	Huhehaote	G	/	170	3	3
The First Hospital Of Huhhot	NC	Neimenggu	Huhehaote	G	/	70	0	0
Fenyang Hospital of Shanxi Province	NC	Shanxi	Fenyang	G	/	70	1	0
The Fifth People's Hospital of Datong	NC	Shanxi	Datong	G	/	52	1	1

The Center Hospital of Shuozhou	NC	Shanxi	Shuozhou	G	/	39	1	1
The Second People's Hospital of Jinzhong	NC	Shanxi	Jinzhong	G	/	31	2	2
Shanxi Maternity and Children Health Hospital	NC	Shanxi	Taiyuan	S	/	131	0	0
Maternal and Child Health Care Hospital of Taiyuan	NC	Shanxi	Taiyuan	S	/	100	0	0
Second Hospital of Jilin University	NE	Jilin	Changchun	G	/	302	4	4
First Hospital of Jilin University	NE	Jilin	Changchun	G	/	116	1	0
Shenyang 242 Hospital	NE	Liaoning	Shenyang	G	/	30	2	2
Anhui Provincial Children's Hospital	EC	Anhui	Hefei	S	/	50	0	0
Wenzhou Central Hospital	EC	Zhejiang	Wenzhou	G	/	102	0	0
Affiliated Yueqing Hospital of Wenzhou Medical University	EC	Zhejiang	Yueqing	G	/	377	0	0
Ningbo No. 1 Hospital	EC	Zhejiang	Ningbo	G	/	319	13	13
The 2nd Affiliated Hospital and Yuying Children's Hospital of WMU	EC	Zhejiang	Wenzhou	G	/	108	0	0
Zhejiang Provincial Hospital of TCM	EC	Zhejiang	Hangzhou	T	/	301	0	0
Shanghai Changzheng Hospital	EC	Shanghai	Shanghai	G	33	67	0	0
Shanghai Ninth People's Hospital Affiliated to Shanghai Jiao Tong University	EC	Shanghai	Shanghai	G	/	118	0	0
Shanghai Minhang District Central Hospital	EC	Shanghai	Shanghai	D/C	/	317	0	0
Shanghai First People's Hospital Branch	EC	Shanghai	Shanghai	D/C	/	159	5	3
Wu Song Central Hospital of Shanghai	EC	Shanghai	Shanghai	D/C	/	139	1	0
Jiangning District Hospital of Traditional Chinese Medicine	EC	Jiangsu	Nanjing	T	/	100	1	0
Yangzhou No.1 People's Hospital	EC	Jiangsu	Yangzhou	G	/	510	0	0
Yantai Municipal Laiyang Central Hospital	EC	Shandong	Laiyang	G	/	100	2	1
Shandong Cancer Hospital	EC	Shandong	Jinan	S	/	435	5	0
Affiliated hospital of Shandong University of traditional Chinese medicine	EC	Shandong	Jinan	T	/	50	0	0
Xintai People's Hospital	EC	Shandong	Xintai	G	/	1001	0	0
Shenzhen Third People's Hospital	SC	Shenzhen	Shenzhen	G	/	481	0	0

The Affiliated Hospital of Hainan medical College	SC	Hainan	Haikou	G	98	386	0/1	0
Zhongshan People's Hospital	SC	Guangdong	Zhongshan	G	/	185	0	0
The Third People’s Hospital of Huizhou	SC	Guangdong	Huizhou	D/C	/	230	1	1
The 44th Hospital of chinese People's Liberation Army	WC	Guizhou	Guiyang	G	/	227	1	1
Bijie First Municipal People's Hospital	WC	Guizhou	Bijie	G	/	303	14	4
Affiliated hospital of Zunyi Medical College	WC	Guizhou	Zunyi	G	/	151	0	0
Guiyang Pulmonary Hospital	WC	Guizhou	Guiyang	S	94	170	0	0
Guizhou Cancer Hospital	WC	Guizhou	Guiyang	S	/	303	3	1
Guiyang Children's Hospital	WC	Guizhou	Guiyang	S	70	217	0	0
The First Affiliated Hospital of Kunming Medical College	WC	Yunnan	Kunming	G	390	819	0/1	0
The Second Affiliated Hospital of Kunming Medical University	WC	Yunnan	Kunming	G	1	507	0/2	0
Traditional Chinese Medical Hospital of Bai Autonomous Prefecture of Dali	WC	Yunnan	Dali	T	16	65	5	0
West China Hospital, Sichuan University	WC	Sichuan	Chengdu	G	/	673	7	7
Sichuan Provincial People's Hospital	WC	Sichuan	Chengdu	G	/	150	0	0
The Third People's Hospital of Chengdu	WC	Sichuan	Chengdu	G	/	1074	1	1
Shuang Liu Hospital of Traditional Chinese Medical	WC	Sichuan	Chengdu	T	/	364	1	1
People's Hospital of Nanbu County	WC	Sichuan	Nanchong	D/C	/	102	1	0
DuJiangyan Traditional Chinese Medicine Hospital	WC	Sichuan	Dujiangyan	T	/	76	0	0
Chongqing Traditional Chinese Medicine Hospital	WC	Chongqing	Chongqing	T	/	603	17	0
The Third People's Hospital of Chongqing	WC	Chongqing	Chongqing	G	/	120	6	1
Chongqing Kaixian County People's Hospital	WC	Chongqing	Chongqing	G	/	323	0	0
Chongqing Three Gorges Central Hospital	WC	Chongqing	Chongqing	G	1	1407	0/8	0/4
The Second Affiliated Hospital of Chongqing Medical University	WC	Chongqing	Chongqing	G	/	158	1	1
Chongqing Jiulongpo First People's Hospital	WC	Chongqing	Chongqing	D/C	/	300	0	0
Lanzhou Petrochemical General Hospital	NW	Gansu	Lanzhou	G	/	97	2	0

Gansu Provincial Hospital of TCM	NW	Gansu	Lanzhou	T	/	301	1	0
People's Hospital of Xinjiang Uigur Autonomous Region	NW	Xijiang	Wulumuqi	G	/	188	1	0
General Hospital of Ningxia Medical University	NW	Ningxia	Yinchuan	G	/	301	3	1
The First People's Hospital of Yinchuan	NW	Ningxia	Yinchuan	G	/	100	5	0
Traditional Chinese Medicine Hospital of the Ningxia Hui Autonomous Region	NW	Ningxia	Yinchuan	T	/	50	0	0
Qinghai Women and Childhood Hospital	NW	Qinghai	Xining	S	/	62	0	0
The First Hospital of Nanchang	CC	Jiangxi	Nanchang	G	/	300	5	3
Jiangxi Provincial People's Hospital	CC	Jiangxi	Nanchang	G	/	435	5	2
Traditional Chinese Medical Hospital of Jiangxi Province	CC	Jiangxi	Nanchang	T	/	252	1	1
The Second Hospital of Nanchang	CC	Jiangxi	Nanchang	T	/	101	1	0
The Fourth Hospital of Changsha	CC	Hunan	Changsha	G	/	135	1	0
The second Xiangya Hospital of Central South University	CC	Hunan	Changsha	G	/	50	1	1
The 152nd Hospital of Chinese People's Liberation Army	CC	Henan	Pingdingshan	G	/	2344	10	4
The First Affiliated Hospital of Zhengzhou University	CC	Henan	Zhengzhou	G	/	151	0	0
Henan Province Hospital of TCM	CC	Henan	Zhengzhou	T	/	302	3	3
Hubei Zhongshan Hospital Affiliated to Wuhan University	CC	Hubei	Wuhan	G	2	101	2	0
Hubei Cancer Hospital	CC	Hubei	Wuhan	S	3	713	0/21	0
Hubei Xinhua Hospital	CC	Hubei	Wuhan	T	346	449	24/15	0
Total cases					2743	27,579	149/285	5/76

a. EC=East China, SC=South China, WC=West China, NE=Northeast, NC=North China, NW=Northwest, CC=Central China

b. G=General Hospital, S=Specialized hospital, T= Traditional Chinese medicine hospital, D/C=District / County hospital

c. R/P: ADR cases in retrospective cohort/prospective cohort

Supplementary Table 3. The distribution of off-label use. Data are n (%).

Item	Product label	Off-label use	Patients with off-label use, N=30,322
Dosage	0-40 ml	>40 ml	22 (0.1)
Diluent	5% glucose or 0.9% normal saline	Beyond 5% glucose or 0.9% normal saline, i.e., fructose injection	2058 (6.8)
Preparation concentration	0-10 %	>10 %	11807 (38.9)
Mode of administration way	Intravenous infusion	Beyond intravenous infusion, i.e., aerosol inhalation.	6 (0.02)
Infusion rate	30–60 drops/min	<30 drops/min	2508 (8.3)
		>60 drops/min	659 (2.2)
Compatibility	Prohibition of being administered with other drugs within a same bottle/ bag	Administered TRQ with other drugs within a same bottle/ bag	173 (0.6)
TCM diagnosis	Disease presenting as wind-warm pulmonary diseases; or syndrome presenting as retention of phlegm-heat in the lung	Syndromes beyond phlegm-heat, phlegm-fire, pyretic pulmonary and wind-heat, or presenting as exterior cold syndrome	852 (2.8)
Diagnosis	Acute upper respiratory infections, pneumonia, or acute exacerbation of chronic obstructive pulmonary disease	Diseases beyond infections and infestations (MedDRA SOC:10006451), and respiratory, thoracic, and mediastinal disorders (MedDRA SOC: 10006458)	6309 (20.8)
Special populations	Be contraindicated in patients with a history of allergy (being allergic to TRQ or alcohol, or having an allergic constitution), hepatic or renal failure, severe pulmonary heart disease with heart failure, pregnant or aged < 24 months	Patients with a history of allergy	2326 (7.7)
		Patients with hepatic or renal failure	413 (1.4)
		Patients having severe pulmonary heart disease with heart failure	12 (0.04)
		Pregnancy women	-
		Infant aged < 24 months	1165 (3.8)
**Total**			20058 (66.1)

Supplementary Table D. Top 10 combined treatments

Treatment subgroup (ATC codes)	Drug Records, N=30055<br>n (%)
Antibacterials for systemic use (J01)	23,105 (76.9)
Blood substitutes and perfusion solutions (B05)	17,254 (57.4)
Cough and cold preparations (R05)	15,847 (52.7)
Drugs for acid-related disorders (A02)	12,107 (40.3)
Drugs for obstructive airway diseases (R03)	11,691 (38.9)
Vitamins (A11)	11,175 (37.2)
Mineral supplements (A12)	9763 (32.5)
Corticosteroids for systemic use (H02)	7650 (25.5)
Analgesics (N02)	7641 (25.4)
Cardiac therapy (C01)	6375 (21.2)
Antithrombotic agents (B01)	5853 (19.5)
Antihemorrhagics (B02)	4793 (16.0)
Drugs for functional gastrointestinal disorders (A03)	4781 (15.9)
Antiviral for systemic use (J02)	4456 (14.8)
Immunostimulants (L03)	4068 (13.5)
Drug used in diabetes (A10)	3982 (13.2)
Diuretics (C03)	3709 (12.3)
Anesthetics (N01)	3556 (11.8)
Calcium channel blockers (C08)	3377 (11.2)

Supplementary Figure A. Distribution of surveillance sites and samples

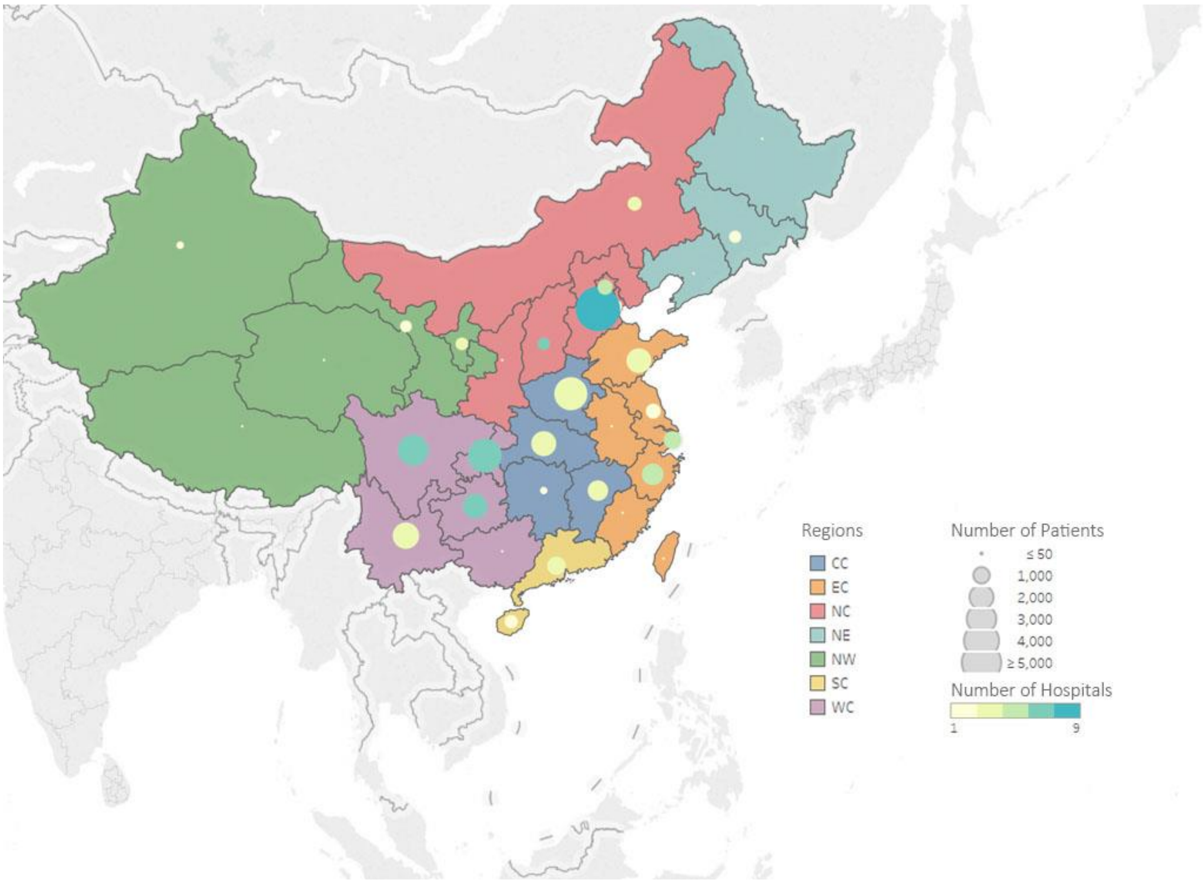

Characteristics of surveillance sites and samples are showed. The colors represents the different regions surveillance sites located. For each circle, the size indicates the number of patients, while the gradation of color presents the number of hospitals in this province.

EC, East China; SC, South China; WC, West China; NE, Northeast; NC, North China; NW, Northwest; CC, Central China.

Supplementary Figure B. The age distribution of the study cohort.

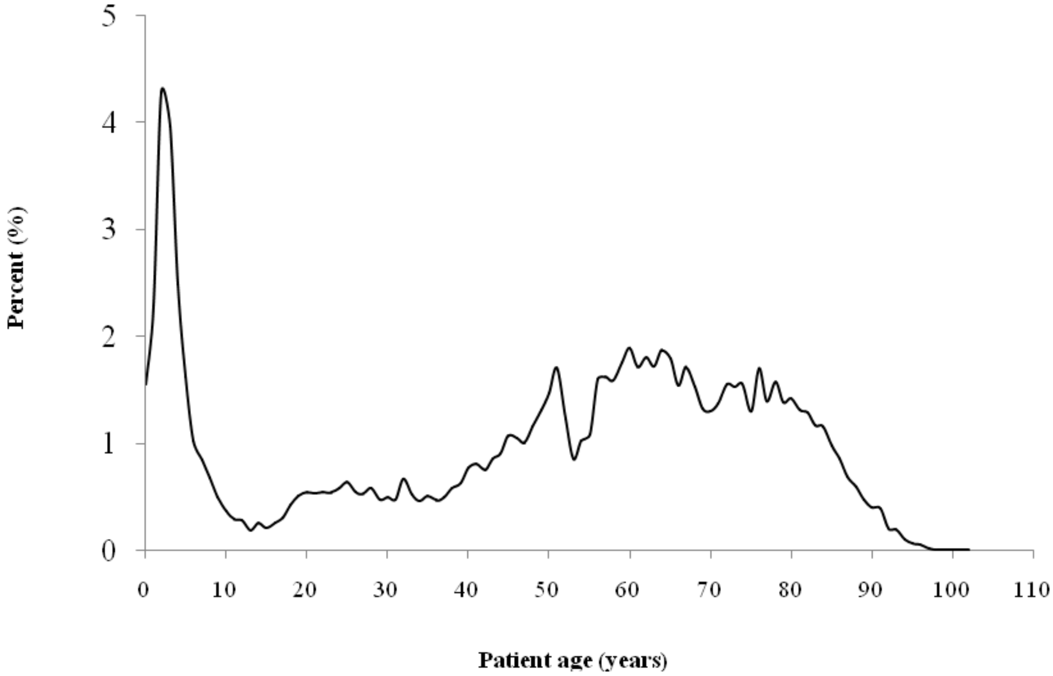

Supplement: Supplementary file 1 [file DataSheet_1.pdf]
